# Unlocking Cognitive Potential: Association of Sarcopenia and Mediterranean Diet on Cognitive Function in Community-Dwelling Elderly of the Dalmatian Region

**DOI:** 10.3390/nu16070991

**Published:** 2024-03-28

**Authors:** Julija Jelaska, Marijana Vučković, Ivana Gugić Ordulj, Ela Kolak, Lucija Šolić Šegvić, Zdravka Đapić Kolak, Irena Keser, Josipa Radić

**Affiliations:** 1 Faculty of Food Technology and Biotechnology, University of Zagreb, 10000 Zagreb, Croatia; jjelaska@gmail.com (J.J.); ivanagugic1979@gmail.com (I.G.O.); 2 Internal Medicine Department, Nephrology and Haemodialysis Division, University Hospital of Split, 21000 Split, Croatia; mavuckovic@kbsplit.hr (M.V.); lucijasolicc@gmail.com (L.Š.Š.); 3 Nutrition and Dietetics Department, University Hospital of Split, 21000 Split, Croatia; elakolak93@gmail.com; 4 Home for the Retired and Elderly Persons Split, 21000 Split, Croatia; zdravkadapickolak@gmail.com; 5 Laboratory for Nutrition Science, Faculty of Food Technology and Biotechnology, University of Zagreb, 10000 Zagreb, Croatia; ikeser@pbf.hr; 6 Department of Internal Medicine, School of Medicine, University of Split, 21000 Split, Croatia

**Keywords:** elderly, cognitive function, sarcopenia, handgrip strength, Mini-Mental State Exam

## Abstract

The aim of this study was to determine the association between muscle strength, adherence to the Mediterranean diet (MeDi) and cognitive function in community-dwelling elderly. General data, data of body composition and anthropometric parameters, clinical and laboratory findings, cognitive test questionnaires (Mini-Mental State Examination—MMSE, Trail Making Test—TMT, Symbol Digit Modalities Test—SDMT), and nutritional assessments (Mini Nutritional Assessment—MNA, Mediterranean Diet Serving Score—MDSS) were obtained for each study participant. Handgrip strength (HS) was used as one of the key parameters for defining probable sarcopenia, among the Short Physical Performance Battery test (SPPB) (for defining physical activity) and the strength, assistance with walking, rising from a chair, climbing stairs, and falls questionnaire (SARC-F). Our cross-sectional study involved 114 participants aged ≥ 60 years, and two-thirds of the participants were female (76.3% vs. 23.7%). Probable sarcopenia was found in 34.7% of them. Using bivariate regression analysis, cognitive deficit among the sarcopenic population was associated with the following groups of collected data: (a) sociodemographic-associated factors—advanced age (OR: 1.07; *p* = 0.004), single marital status (OR: 3.25; *p* = 0.03), and low level of education (OR: 0.22; *p* < 0.003); (b) behavioral-associated factors—duration of institutionalization (OR: 1.05; *p* = 0.007), performance of heavy physical work (OR: 6.26; *p* = 0.001), low physical activity (OR: 0.08; *p* = 0.002), and risk of malnutrition (OR: 3.87; *p* = 0.005); (c) disease-related factors—loss of appetite (OR: 2.24; *p* = 0.04), information processing speed (OR: 0.88; *p* < 0.001), blood pressure systolic/diastolic variables (OR: 0.96/0.96; *p* = 0.002/0.02), medications (OR: 1.19; *p* = 0.005), predictive sarcopenia score ≥ 4 (OR: 3.1; *p* = 0.003), and low muscle strength (OR: 0.92; *p* = 0.002). Cognitive preservation among the sarcopenic population was associated with married status (OR: 0.23; *p* = 0.20), a high level of education (OR: 0.18; *p* = 0.002), smoking (OR: 0.33; *p* = 0.02), high physical activity (OR: 0.07; *p* < 0.001), and dietary habits using poultry (OR: 0.12; *p* = 0.004). The results suggest a significant association between sarcopenia and cognitive function in community-dwelling elderly, highlighting the need for regular nutritional interventions in this special population.

## 1. Introduction

The extension of human life over the years also increases the need for nutritional intervention in the elderly, not only because of numerous comorbidities that may occur during life but also because of the physiological ageing of the organism. The prevalence of protein-energy deficiency among older people living in community-dwelling centers is 15–38% [1]. The occurrence of malnutrition in this population, as a state of nutrient deficiency, leads to a measurable change in body functions and physiological effects, which consequently have a negative impact on cognitive functions. Individuals in the fifth decade of life experience a loss of lean muscle mass and consequently of strength of 0.8 and 1–3% on average due to the physiological function of aging [2]. The overall prevalence rate of sarcopenia in older adults living in a community range from 0.4 to 9.3% according to the European Working Group on Sarcopenia in Older Persons (EWGSOP) classification. EWGSOP defines the condition of aging and the numerous associated acute and chronic conditions that occur during the lifetime of any individual and are associated with a progressive loss of muscle mass, strength, and muscle function as a diagnosis of sarcopenia. To make the appropriate diagnosis, two criteria must be met: decreased muscle mass and decreased muscle strength and/or decreased physical performance [3].

According to the findings of a recent systematic review, individuals with sarcopenia were roughly twice as likely to experience dementia and mild cognitive impairment [4]. This correlation held true for several study populations, study regions, definitions of sarcopenia, definitions of cognitive impairment, and study quality levels [4]. Neural and vascular adaptations are thought to improve cognitive function by enhancing neurogenesis, angiogenesis, and synaptic plasticity; reducing proinflammatory processes; and decreasing cellular damage from oxidative stress through improved muscle mass status and consequently muscle function [5].

Among different dietary patterns, the Mediterranean diet (MeDi) has been recognized as a dietary pattern that significantly influences an individual’s cognitive and nutritional status. The analysis of available studies has demonstrated consistent evidence of the protective effect that the MeDi has against cognitive decline in older people, as it combines foods and nutrients that are potentially protective and beneficial against cognitive dysfunction (polyphenols from fruits and vegetables, oleic fatty acid from olive oil, omega-3 fatty acids from fish oil, vitamin B12, folic acid, and antioxidants). Their regular consumption has been associated with improved cognitive performance, better memory, and a reduced risk of developing neurodegenerative diseases such as Alzheimer’s disease [6,7,8,9]. Detailed knowledge of the mechanisms linking the MeDi and cognition in elderly people is not yet known, and RCTs relating to the MeDi in this specific population are lacking. Moreover, the MeDi has proven to be beneficial in maintaining nutritional status. The abundance of diverse nutrient-rich foods in this diet provides adequate amounts of vitamins, minerals, and antioxidants that are crucial for proper bodily functions. Furthermore, the MeDi has a preventive effect on the development of diseases and conditions such as cardiovascular disease, diabetes, hypertension, and metabolic syndrome [10,11,12]. The MeDi has been included in the world list of intangible cultural heritage (under UNESCO, 2013), and in the region of Dalmatia, it is influenced by the ecological, climatology, historical, and cultural factors of the Mediterranean [13].

We hypothesize that higher cognitive decline is associated with higher levels of sarcopenia and lower levels of MeDi adherence. The aim of this study was to determine the association between sarcopenia, MeDi adherence, and cognitive deficits in community-dwelling older people in the Dalmatian region. Additionally, we seek to compare two groups of participants based on the presence or absence of cognitive deficits.

## 2. Materials and Methods

This cross-sectional study examined, in a sample of 114 community-dwelling older people, the impact of the presence of probable sarcopenia (we combined measures of grip strength and physical performance with body composition and predictive sarcopenia score) and its direct association with cognitive impairment on the following key areas: (1) the detection of cognitive impairment, dementia and monitoring of condition over time, acquisition of basic data on temporal and spatial orientation, recall of basic concepts, ability to perform basic arithmetic operations, writing, reading, and tracing slightly more complex geometric figures—Mini-Mental State Examination (MMSE) [14]; (2) the assessment of the knowledge of the ability to think, remember, and reason for the purpose of detecting dementia—Trail Making Test (TMT) [15,16,17,18]; (3) the assessment of the speed of information processing, as the latest adaptation of the subscale of the Wechsler Intelligence Test called Encoding—Symbol Digit Modalities Test (SDMT) [19].

### 2.1. Study Population and Eligibility Criteria

The inclusion criteria were as follows: (1) participants: older people ≥ 60 years old living in a community-dwelling center, with no gender or nationality restriction; (2) those who were willingly participated in the study.

The exclusion criteria were as follows: (1) patients with an acute illness and metastatic active malignant disease; (2) with a hospital stay of more than 3 months; (3) with a diagnosis of progressive brain disease characterized by loss of memory, thinking ability, and personality changes (Alzheimer’s disease, dementia); (4) on psychotropic drug therapy; (5) immobile, amputee, or implanted endoprosthesis; (6) implanted stent or pacemaker; (7) without written informed consent to participate.

Among the total of 206 participants who had a clinic visit, 60 of them were immediately excluded from the study due to limitations that were necessary to conduct testing of mental and physical abilities. In the end, 146 subjects took part in the testing. Among them, 114 subjects met the next key required criteria for defining specific cognitive deficits and muscle status, which we decided to examine.

**Figure 1:**
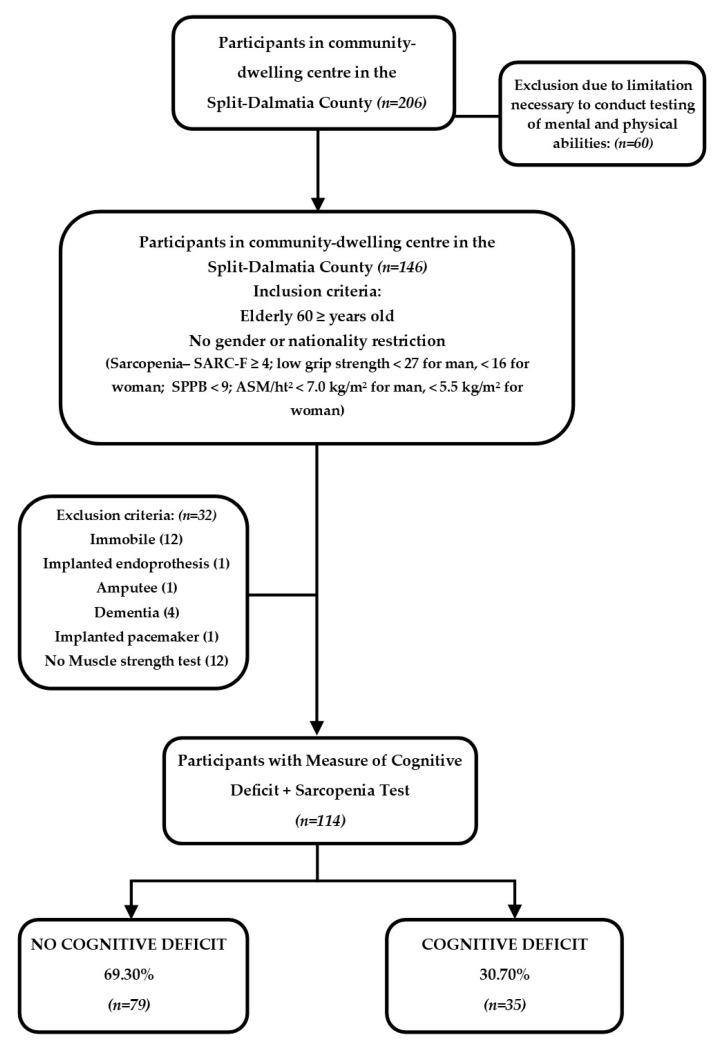
Study design. Abbreviations: SARC-F—the strength, assistance with walking, rising from a chair, climbing stairs, and falls questionnaire; MMSE—Mini-Mental State Examination.

The study flowchart is shown in Figure 1.

All respondents were placed in a home for the elderly and infirm. General demographic data, presence of chronic diseases, and nutritional risk factors were collected from medical records and questionnaires filled out by research staff in conversation with the patient. The values of the serum levels of the biochemical parameters studied were collected from laboratory blood results available in the medical records.

For this purpose, basic data were collected: age, sex, marriage status, previous job activity, age of entering the institution, treatment for arterial hypertension, surgical interventions, duration of hospitalization and episodes of acute illness in the past year, prescribed medical therapy, harmful habits, weight changes at least 6 months prior to the study, and other symptoms related to nutritional status.

Anthropometric parameters and body composition parameters were assessed.

Moreover, laboratory parameters including serum levels of hemoglobin (g/L), mean corpuscular volume (MCV) (fL), creatinine (µmol/L), urea, plasma glucose (mmol/L), albumin (g/L), total cholesterol, low-density lipoprotein (LDL) cholesterol, triglycerides, potassium, calcium, phosphorus (mmol/L), and uric acid (µmol/L) were recorded. In addition, the albuminuria values (mg/day) were noted and the albumin/creatinine ratio in the urine and the glomerular filtration rate were calculated, which were taken from the patient’s medical history. Peripheral systolic (SBP) and diastolic blood pressure (DBP). was measured with a sphygmomanometer: three measurements were taken, and the average value was determined. The subject was in a seated position in a quiet environment, and the right upper arm was measured according to the size of the arm circumference).

Validated questionnaires were used to assess nutritional status, predict cognitive deficits, assess cognitive abilities of thinking, memory, and reasoning in dementia research, and assess processing speed.

### 2.2. The Assessment of Sarcopenia

This research, following the European consensus on the definition and diagnosis of sarcopenia, describes the diagnostic procedures that were used to assess the presence of reduced muscle mass, muscle strength, and/or physical performance and their direct association with cognitive impairment.

To define the diagnosis of sarcopenia, data were obtained to calculate the parameter that summarizes the affected area of decreased muscle mass (appendicular skeletal muscle mass—ASM) [20]. ASM was quantified by summing the lean muscle mass of both arms and legs. The square of the height (ASM/ht^2^) was used to compare the muscle masses of the participants. The Tanita MC-780 Multi Frequency Segmental Body Analyzer (Tanita, Tokyo, Japan) using bioelectrical impedance analysis technology was used to determine muscle mass.

A dynamometer (Saehan, Hwaseong-si, Republic of Korea) handgrip test was used to assess muscle strength. To measure physical activity, the SPPB test was used. The strength, assistance with walking, rising from a chair, climbing stairs, and falls questionnaire (SARC-F) questionnaire was used to define predictive sarcopenia.

Using the EWGSOP^2^ guidelines, low muscle strength was used as one of the key parameters for defining probable sarcopenia, among the low performance of the SPPB test (for defining physical activity) and the SARC-F questionnaire score. Sarcopenia was confirmed when both low HS and/or slow gait speed as part of the low physical performance test (SPPB) and low percentage muscle mass (ASM/ht^2^) were below cut-off points. In both EWGSOP 1 and EWGSOP 2, low muscle mass was defined as an ASM/ht^2^ of <7.0 kg/m^2^ for men and <5.5 kg/m^2^ for women. Low grip strength was defined as a maximum grip strength of <27 kg for men and <16 kg for women. Low physical performance on the SPPB test was defined as a total score of <9 points for men and women [21,22]. The SPPB consists of three assessments: an evaluation of standing balance using three different foot positions, a timed walk at the usual elderly pace, and the task of standing up and sitting down from a chair five times consecutively. In the balance assessment, participants were required to maintain specific foot positions for 10 s each. The gait speed test involved measuring the time taken to cover a 4 m distance at a regular pace, with two repetitions and the shorter time used for analysis. During the chair test, participants had to stand and sit five times quickly with crossed arms, only after demonstrating the ability to stand without using their arms. Scores ranging from 0 (worst performance) to 4 (best performance) were assigned for each test. The balancing test was scored based on a hierarchical combination of performance in three positions, while a score of 0 was given for the other two tests if the participant did not attempt or complete them. Scores of 1–4 were assigned for the timed tests. Furthermore, a total score for the entire battery was calculated by summing up the scores of all three tests, ranging from 0 to 12. The SARC-F questionnaire, as a simple, rapid test to diagnose sarcopenia, was used to evaluate functional ability, assessing five aspects: strength, assistance in walking, rising from a chair, climbing stairs, and occurrences of falls. Responses were graded on a scale from 0 to 2 points, and the cumulative score, ranging from 0 to 10, was recorded based on the total points obtained. The total score ≥ 4 was predictive of sarcopenia [23].

### 2.3. Assessment of the Cognitive Deficit

To assess cognitive deficit for the early detection of cognitive impairment and dementia and monitoring of the condition over time, the MMSE test was used to obtain basic information on orientation in time and space, attention, memory, ability to perform basic arithmetic operations, language, and visual–spatial skills. Scores from 24 to 30 refer to no cognitive impairment, 18 to 23 mild cognitive impairment, and 0 to 17 severe cognitive impairment [24]. The TMT has been used to cognitively assess thinking, memory, and mind for the purpose of detecting dementia. It includes Test Part A and Test Part B. Both are reported as the number of seconds required to complete the task (higher scores reveal greater impairment). Trail A > 78 s and Trail B > 273 s are defined as deficient [18,25]. The SDMT is a letter substitution test, the final subscale of the Wechsler intelligence test called Coding, which tests information processing speed. Using a reference key, the respondents had 90 s to pair specific numbers with given geometric figures (max = 110) [26]. The final categorization of the participants was performed based on the MMSE score, which correlates with TMT-A, TMT-B, and SDMT tests.

### 2.4. Assessment of Nutritional Status

A rapidly validated method of assessing nutritional status, the MNA, assesses the risk of malnutrition in older people to ensure early nutritional intervention if needed. MNA is a method using simple measurements and short questions. A score of 24–30 indicates a well-nourished person and needs no further intervention. A score of 17–23.5 indicates a person at risk of malnutrition, and in the case of no weight loss demands only monitoring, but those with detected weight loss need nutrition intervention (diet and oral nutritional supplementation). A score <17 indicates a malnourished person and requires mandatory nutritional intervention [27,28]. Basic anthropometric measurements included recording body weight (kg), height (cm), waist circumference (cm), hip circumference (cm), and upper arm circumference (cm). The Tanita MC-780 Multi-Frequency Segmental Body Composition Analyzer (TANITA, Tokyo, Japan) was used to measure body composition. A variety of data were gathered, including total body water, fat-free mass, visceral fat level, fat percentage, fat mass (kg), and predicted muscle and bone mass (kg).

Body mass index (BMI) and waist-to-hip and waist-to-height ratios (WHtR) were calculated from the above data. A flexible plastic centimeter tape was used to measure the circumference of the upper arm, waist, and hip. BMI less than 18.5 was defined as underweight, a range between 18.5 and 24.9 refers to normal weight, 25 and 29.9 to overweight, and 30 or more is considered obese [29]. Age-standardized mean waist circumference ranges between populations from 83 to 98 cm in men and from 78 to 91 cm in women. Hip circumference ranged from 94 to 105 cm and from 97 to 108 cm in men and women, and mean WHtR ranged from 0.87 to 0.99 and from 0.76 to 0.84 [30]. General data on weight loss, lifestyle, medication, and mobility were recorded. Information was recorded on the number of meals, food and water intake, and the ability to eat independently, as well as a self-assessment of participants’ own health and nutritional status.

### 2.5. Assessment of Mediterranean Diet (MeDi)

The Mediterranean Diet Serving Score (MDSS) questionnaire was used to consolidate data on eating habits in clinical practice. It is based on food consumption and food representation groups per meal, day, and week, divided into fourteen groups. The score is given according to the new Mediterranean food pyramid as follows: 3 points for fruits, vegetables, olive oil, and cereals consumed with each meal; 2 points for dairy products and nuts if consumed daily; and 1 point for the recommended number of servings per week for potatoes (≤3), legumes (≥2), eggs (2–4), fish (≥2), poultry (2), red meat (<2), sweets (≤2), and fermented beverages (1–2 glasses a day) [31]. The MDSS ranges from 0 to 24 points for adults. A score ≥14 on the MDSS scale is considered to adhere to the principles of the MeDi [31].

### 2.6. Statistical Analysis

Categorical data are represented by absolute and relative frequencies. Numerical data were described by the median and the limits of the interquartile range. Differences of categorical variables were tested by the Chi-square test and by Fisher’s exact test. The normality of the distribution of numerical variables was tested by the Shapiro–Wilk test. Differences between the two independent groups were tested by Mann–Whitney’s U test. Differences in numerical variables in cases of 3 or more groups were tested by the Kruskal–Wallis test. Spearman’s Rho test was used to determine the association between non-normally distributed variables. Logistic regression analysis was used to analyze the independent factors associated with the likelihood of malnutrition. The significance level was set to Alpha = 0.05. The statistical analysis was performed using MedCalc^®^ Statistical Software version 22.006 (MedCalc Software Ltd., Ostend, Belgium; https://www.medcalc.org, accessed on 15 February 2023) and SPSS ver 23 (IBM Corp. Released 2015. Armonk, NY, USA).

## 3. Results

### 3.1. Characteristics of the Study Population

Among a total of 114 participants, 87 (76.3%) were female, with a mean age of 81 years (IQR 74–86).

#### Study Population by Cognitive Decline Due to MMSE

The study cohort was stratified into two categories based on their MMSE score. Those participants with an MMSE score ≥ 24 were categorized as exhibiting no cognitive decline (*n* = 79; 69.30%), while those scoring <24 were considered to have moderate-to-severe cognitive decline (*n* = 35, 29.82%). Table 1 highlights significant differences between these two groups regarding the manifestation of cognitive decline.

Participants with moderate or severe cognitive decline determined by the MMSE were statistically significantly older (*p* = 0.001).

No significant differences were observed in gender concerning the presence of cognitive decline.

Regarding the sociodemographic characteristics, there are significantly more participants in the group with cognitive decline who were divorced or widowed, according to their marital status (*p* = 0.02), who had less than a high school education (*p* = 0.001) and engaged in heavier physical activity (*p* = 0.03).

In terms of smoking habits, participants with cognitive decline smoked more cigarettes for a longer duration (*p* = 0.01).

In terms of clinical and biochemical parameters, individuals with cognitive decline exhibited significantly lower values for both SBP (*p* < 0.001) and DBP (*p* < 0.001) compared to participants without cognitive decline. Notably, the mean SBP and DBP values for both groups fell within the normal range (139/80 mmHg in the no cognitive decline group vs. 121/72 mmHg in the cognitive decline group). Nevertheless, the presence of arterial hypertension was not different between the groups.

Furthermore, participants with cognitive decline were prescribed significantly more different medications than participants with no cognitive decline (*p* = 0.02). When it comes to biochemical parameters, participants with cognitive decline showed higher values of total cholesterol (*p* = 0.05), triglycerides (*p* = 0.04), and serum calcium levels (*p* = 0.02) than participants with no cognitive decline.

The nutritional status of study participants and differences between the two groups of participants according to MMSE score are shown in Table 2. No significant differences in parameters were observed when comparing two groups of subjects.

Differences in sarcopenia-predicting parameters regarding the presence of cognitive decline assessed by MMSE are shown in Table 3.

The results of the overall tests of the association of sarcopenia and cognitive impairment are shown in Table 3 (only statistically significant differences are shown). A total of 99 subjects managed to complete the SPPB test. Participants with cognitive decline according to the SPPB test had worse physical performance (*p* < 0.001), had lower muscle strength (*p* < 0.001), lower MNA score (*p* = 0.01), and a higher probability of sarcopenia according to SARC-F (*p* = 0.006), while the predicted muscle mass (kg) was not shown to be statistically different (*p* = 0.57).

A significant difference was found for all tests applied (Supplementary Table S3).

Table 4 (only statistically significant correlations are shown) shows the results of the association between each individual variable, separately, and cognitive impairment by the MMSE.

**Figure 2:**
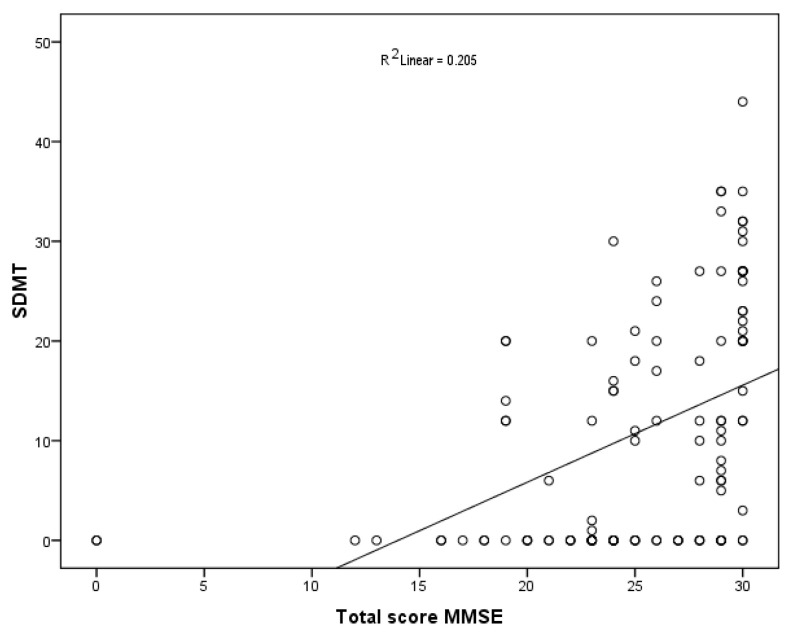
Correlation between the total score of the Mini Mental State Examination (MMSE) and the Symbol Digit Modalities Test (SDMT) (Scatterplot with representation of trend line and determination coefficient R^2^; Each circle represents the value of SDMT for a specific MMSE).

Higher values of MMSE score were associated with higher muscle strength (Rho 0.438; *p* < 0.001), SBP (Rho 0.364; *p* < 0.001), DBP (Rho 0.254; *p* = 0.01), upper arm circumference (Rho 0.342; *p* = 0.05), MNA total score (Rho 0.206; *p* = 0.03), TMT-A (s) (Rho 0.326; *p* < 0.001), TMT-B (s) (Rho 0.517; *p* < 0.001), and SDMT (Rho 0.569; *p* > 0.001) (Table 4) (Figure 2).

MMSE score was inversely associated with age (Rho −0.286; *p* < 0.001), age of entering the institution (Rho −0.306; *p* < 0.001), number of medications (Rho −0.218; *p* = 0.03), total MDSS score (Rho −0.177; *p* = 0.06), and among those in the groups that consumed wine (Rho −0.198; *p* = 0.03) (Table 4).

In the additional table, individual groups of food that may affect the outcome are presented (see Supplementary Table S1).

In Table 5 (only statistically significant *p* are shown) observing which predictor significantly affects the decline of cognitive functions, the independent variables are presented.

Certain parameters have been found to have an impact on decreasing cognitive decline: (1) higher educational level compared to low education level (OR: 0.18; Cl95% 0.06–0.54; *p* = 0.002; ß = −1.72); (2) smoker (OR: 0.33; Cl95% 0.13–0.83; *p* = 0.02; ß = −1.09); (3) higher muscle strength (OR: 0.92; Cl95% 0.89–0.97; *p* = 0.002; ß = −0.08); (4) SBP deviation from reference values (OR: 0.96; Cl95% 0.94–0.98; *p* = 0.002; ß = −0.03); (5) high physical activity compared to low physical activity (OR: 0.07; Cl95% 0.02–0.34; *p* = 0.001; ß = −2.62); (6) SDMT test for the speed of processing information (OR: 0.88; Cl95% 0.83–0.93; *p* < 0.001; ß = −0.13). As shown, they act protectively, OR < 1, reducing the probability of a decrease in cognitive function.

The other parameters, which have shown significance in the development of deterioration, we can interpretate as follows: older participants had a 1.07 times higher chance for the development of the deficit (OR: 1.07; Cl95% 1.02–1.12; *p* = 0.004; ß = 0.07); higher age of entering the institution results in a 1.05 times higher chance for cognitive decline (OR: 1.05; Cl95% 1.01–1.09; *p* = 0.007; ß = 0.05); widowed compared to unmarried participants had a 3.25 times higher chance for the development of cognitive deficit (OR: 3.25; Cl95% 1.12–9.46; *p* = 0.03; ß = 1.18); those who performed heavy physical work showed a 6.26 times higher possibility for lower values of MMSE compared to sedentary activity (OR: 6.26; Cl95% 2.07–18.9; *p* = 0.001; ß = 1.83); a loss of appetite resulted in a 2.24 times increased chance of deficit (OR: 2.24; Cl95% 1.01–4.94; *p* = 0.04; ß = 0.80); more medications through day therapy resulted in 1.19 times the chance of deterioration (OR: 1.19; Cl95% 1.05–1.34; *p* = 0.005; ß = 0.17); those at risk of malnutrition compared to malnourished subjects had a 3.87 times higher chance for higher levels of cognitive decline (OR: 3.87; Cl95% 1.52–9.89; *p* = 0.005; ß = 1.365); those with probable sarcopenia had a 3.1 times greater chance for deficit (OR: 3.1; Cl95% 1.48–6.49; *p* = 0.003; ß = 1.13); a higher triglycerides value resulted in 3.05 times higher probability for deterioration (OR: 3.05; Cl95% 1.08–8.6; *p* = 0.03; ß = 1.12); and non-compliance with nutritional recommendations showed an increase in cognitive deficit (OR: 1.1; Cl95% 0.96–1.26; *p* = 0.16; ß = 0.09).

In the additional table, individual groups of food are specified which affect cognitive decline (see Supplementary Table S2).

Potential predictors for explaining the MMSE (logistic stepwise multivariate regression adjusted for age and gender) are shown in Table 6.

The model is consistently significant (χ^2^ = 46.4; *p* < 0.001) and explains between 41% (Cox and Snell R^2^) and 75% (Negelkerke) of the variance in greater cognitive deficit. Significant predictors of greater cognitive deficit include the older age of the respondents (OR = 2.29) and predictive sarcopenia (≥4) (OR = 353.6).

## 4. Discussion

In this cross-sectional study involving 114 community-dwelling older adults, with no restrictions on testing mental and physical abilities, we demonstrated the impact of two crucial factors, sarcopenia and MeDi adherence, on cognition. To our knowledge, this is the first study to evaluate the association of sarcopenia and MeDi adherence with cognitive function in community-dwelling elderly in this region. This research highlights the identifiable predictive factors that are associated with nutritional status parameters and decline in cognitive ability in community-dwelling elderly.

Our findings suggest that the early detection of malnutrition may allow for timely intervention and thus preserve the cognitive abilities of older people.

Specifically, the study investigated the association between affected nutritional status due to poorer MeDi adherence and the presence of muscle weakness, indicative of probable sarcopenia, as well as the occurrence of cognitive deficits.

More limited subjects, regarding mobility and limitation in physical performance, experience negative nitrogen balance because of inactivity. Those with a reduced protein intake, i.e., non-adherence to MeDi, experienced a loss of muscle mass, and in combination with a loss of muscle strength and/or physical performance, they exhibited an increase in the cognitive deficit. Those subjects were categorized as having probable sarcopenia. Cognitive functions were assessed using the MMSE test. In accordance with previous research, a significantly higher cognitive deficit is observed in the older population as our results suggest [6,7,8,9]. Additionally, no significant deviations were found between the two groups of participants based on gender, aligning with existing research [32].

In our study, marital status showed a significant negative correlation with cognitive decline. Conversely, individuals who are single (divorced/widowed) tend to experience a negative cognitive status associated with various health issues such as poor sleep, depression, and socialization challenges [33].

Research indicates that older individuals experience many challenges of a personal, physical, and social nature, leading to increased vulnerability. This population tends to participate less in social activities, reduce social contacts, and significantly decrease physical activity, resulting in compromised health and a more pronounced cognitive deficit [34].

The level of education potentially has a positive impact on cognition. Our results were generally uniform across various studies. One study has shown that MMSE scores decline in older adults and particularly whether individuals with fewer years of formal education are likely to decline more rapidly [35]. The other study has shown that, on average, MMSE scores exhibited a decline over time, particularly among older individuals. Education was a predictor of MMSE scores, although, with two exceptions, it did not show a correlation with the decline in MMSE over time [36].

Evidence suggests that engaging in physical activity leads to enhanced cognitive function and/or a reduced risk of mild cognitive impairment (MCI). This is in accordance with other studies which associate physical activity and cognition [37]. Physical activity is linked to preservation on neuronal connectivity and positive alterations in neurogenesis [38,39]. Physical changes in the body brought on by physical exercise provide the basis for physiological mechanisms such as increased cerebral blood flow, altered arousal levels, alterations in neurotransmitter release, and structural changes in the central nervous system [40].

The initial identification of potential mechanisms underlying the relationship between physical activity and cognition included a reduction in depressive symptoms and the enhancement of sleep quality [41].

Due to smoking habits, this study has shown that heavy smoking has been associated with cognitive impairment and a decline in cognitive function. This may be because the different neurobiological mechanisms come into play during acute and chronic smoking. The extensive presence of nicotinic acetylcholine receptors (nAChRs) throughout the brain impacts various neurotransmitter systems (norepinephrine, serotonin, and dopamine). So, nicotine affects a broad spectrum of cognitive functions, including sensory perception, motor skills, attention, executive function, learning, and memory [42].

Our participants’ mean values for SBP and DBP, for both groups’ (no cognitive decline vs. cognitive decline) results were within the normal range, but those with cognitive decline had significantly lower values for both SBP and DBP. A study by Momtaz et al. of 1067 community-dwelling older adults found hypotension to be negatively associated with cognitive function in accordance with our results [43].

It is speculated that, in cases of a large drop in blood pressure brought on by several antihypertensive medications, abnormal circulatory autoregulation may result in tissue hypoperfusion in the setting of extreme frailty. Therefore, in these patients, a response to therapy that results in an SBP < 130 mmHg may enhance rather than decrease morbidity and death [44].

The presence of arterial hypertension was not different between the two groups. Due to the significant heterogeneity among individuals concerning the functional status, weaknesses, and autonomy of older individuals, a uniform strategy cannot be applied.

For a profile of functional loss, preserving daily life activities requires a more detailed geriatric assessment to define adjustments in various therapeutic strategies.

The results from our study suggest a higher risk of cognitive decline associated with a greater number of prescribed medications and this is supported by previous studies. Older adults commonly have been prescribed with chronic therapy with anticholinergic medication, such as antihypertensive agents, antihistamines, antispasmodics, antidepressants, and therapy for a short period for treating infections, pain, and constipation [45]. The probability of short-term cognitive decline in older people increases significantly with the use of anticholinergics. This risk persists even if antipsychotics are excluded from the analysis, although the adverse effects remain the same [46].

The presented higher values of total cholesterol (TC) and triglycerides support the cholesterol–cognition association. Both parameters were significantly higher among the participants with cognitive decline. In some other studies, an association between cholesterol profiles and the risk of cognitive decline among older adults was inconclusive, but a significant correlation was found between incremental TC, LDL-cholesterol (LDL-c), and a slower annual decline in MMSE score [47]. Higher cholesterol levels were reported to have an association with increased risk of dementia indirectly, via inducing atherosclerosis and impairing blood flow [48]. In another study, a modest rise in serum LDL-c levels was linked to enhancements in visual and executive abilities, language, memory, and delayed recall, which may be advantageous for cognitive function in older adults. Higher levels of circulating TC and high-density lipoprotein cholesterol (HDL-c) were associated with diminished cognitive function [49].

Although numerous studies have shown a correlation between higher BMI and an increased risk of cognitive impairment [50,51], findings from alternative studies suggest that excess weight, obesity, and central adiposity could potentially be a protective factor against cognitive impairment and dementia in the elderly [52]. In our study, no connections were found among the two groups of participants, whether with or without cognitive deficits, concerning BMI and body composition. There was no significant difference in parameters when comparing these two groups of subjects. In another study, which analyzed cognitive function and body composition for subjects aged 65 years and older using the Korean MMSE and dual-energy X-ray absorptiometry (DEXA), results indicated that higher fat mass and lean body mass were associated with a lower risk of cognitive impairment in older women [53]. In a recent study examining the longitudinal effects of alterations in body composition on cognitive function in community-dwelling adults, over a 6-year period, the results revealed that a reduction in fat-free mass and muscle mass is associated with an accelerated cognitive decline in men. However, conversely, no significant association was observed in women [54].

Sarcopenia may be the critical predictor of declining cognition, which refers to the age-related loss of muscle mass and function. A result from a recent meta-analysis revealed that there exists a connection between cognitive impairment and sarcopenia [55]. Also, basic research suggests that inflammatory markers and hormonal pathways (interleukin-6, C-reactive protein, myokines, and serum testosterone) play a role in the association between sarcopenia and cognitive impairment. They proposed that pathogeneses explain the link between sarcopenia, obesity, and cognitive dysfunction, including chronic inflammation, adipose tissue dysfunction, oxidative stress, insulin resistance, and mitochondrial dysfunction, all of which are age-related [56,57].

The results of our study on the examinations evaluating the relationship between sarcopenia and cognitive impairment show that those who exhibit cognitive decline based on the SPPB test have poorer physical performance, lower muscle strength, higher malnutrition status, and an increased likelihood of sarcopenia as indicated by the SARC-F test.

However, no statistically significant difference in predicted muscle mass (kg) was observed. The SPPB results we observed are in line with other studies, such as a recent study that found a positive association between a higher MMSE score and a lower duration of 4 m walking and a higher HS load [58]. Regarding predicted muscle mass (kg), our finding is in contradiction with previous research suggesting an association between poorer muscle status and worsening cognition. This suggests the need for additional studies to investigate the cases in which the coexistence of preserved muscle mass and poor cognitive performance is observed.

Several studies have documented significant connections between cognitive performance and HS in community-dwelling older adults [24,59]. Worse scores of HS showed an association with cognitive decline, suggesting that grip strength could be an early indirect marker of forthcoming cognitive deterioration [60]. These findings are consistent with our results. Naharachi et al. in their study showed that an elevated blood pressure index (BPI; BPI = SBP/DBP) is significantly linked to cognitive difficulties in performing MMSE, even after adjusting for potential confounding factors [61].

Guo et al. investigated the correlation between blood pressure and cognitive function, assessed by the MMSE, in a community-based Swedish cohort. Multiple linear regression analysis revealed a positive and significant association between both the SBP and DBP measured. They suggest that maintaining a specific blood pressure level, particularly a SBP of at least 130 mmHg, may be crucial for preserving cognitive function in the very old [62]. Other studies have shown that individuals with elevated SBP face an increased risk of cognitive decline [63]. Contrary to that, our study revealed that participants with lower SBP exhibit a greater cognitive deficit. Considering all this, it is crucial to note that the average SBP and DBP of both the participants in our study who had cognitive impairment and those who did not fall within the normal range. As such, the findings should be interpreted accordingly.

We also investigated the association between lower MMSE scores and the risk of malnutrition, assessed by the MNA score. Our findings indicate that a higher MNA total score is associated with the preservation of cognition in older adults. Xiaolei et al. also indicated that nutritional status mediates the association between cognitive decline and sarcopenia. Thus, they propose that sustaining a favorable nutritional status can postpone the adverse impacts of cognitive decline and consequently the incidence of sarcopenia [64].

In our study, the obtained results demonstrated consistency between the TMT parts A and B and the SDMT test with MMSE results. It was also revealed that a greater number of participants with lower MMSE scores were able to complete the TMT-A test compared to the TMT-B and SDMT tests. It is in accordance with another study which showed that the MMSE score, and age exhibited a strong correlation with performance in both parts TMT A and TMT B. These findings also imply that the TMTs could serve as effective tools for identifying age-related alterations in attention and executive function before they clinically manifest [65].

The MeDi, preferred in the participant’s region, is abundant in nutritionally rich foods. Its ingredients provide an adequate amount of vitamins, minerals, and antioxidants, which play a crucial role in preserving the metabolism and proper physical functioning of the organism. Non-compliance with nutritional recommendations demonstrated an increased association with cognitive deficit [66].

Individuals with poorer dietary intake are at risk of malnutrition and had a significantly higher chance of experiencing elevated levels of cognitive decline. Protein-energy malnutrition (PEM) affects the immune system in older individuals on several pathways: decreased antibody production, interleukin release, and gut immune barrier function. Over the life cycle, there is a decline in the mass of immune tissue, and immune aging is related to an immunodeficiency state characterized by decreased proliferation of T lymphocytes. Both PEM and aging underscore the negative effects of impaired nutrition upon immunocompetence [67]. This study has shown that an elevated lipid level, associated with an inadequate macronutrient ratio, was associated with a higher probability of deterioration. The subjects who adhered to the MeDi were provided with the proper ratio of monounsaturated (olive oil) or polyunsaturated (soybean oil, fish oil…) fatty acids. Those “healthy” fats are energy-dense and act protectively on vascular health as well contributing to the preservation of energy balance. Although the mechanism of benefits of the MeDi is not fully understood, it is well known that several signal pathways for the reduction in oxidative stress, neurological preservation, immunosuppression of inflammation, and a rich and balanced intestinal microbiome for the proper function of digestion and metabolism are activated [68]. Despite aligning with numerous observations and findings from previously presented studies, our data, relying on independent variables, revealed several individual predictors that significantly impact cognition: loss of appetite, increased medication intake, risk of malnutrition, probable sarcopenia, higher triglyceride levels in laboratory findings, and poorer adherence to the MeDi. After performing a multivariate regression analysis, the advanced age of the respondents and the presence of predictive sarcopenia were identified as predictors of cognitive decline. These two indicators emphasize the need for early nutritional intervention to prevent diseases that are often associated with the aging process.

This study has some limitations. Due to the cross-sectional design, we cannot draw causal conclusions. We included a smaller number of participants, but it is still a representative sample. This is because we had to exclude a certain number of participants due to their advanced age, which limited their ability to perform cognitive tests and physical performance. In this cross-sectional study, we were able to collect only limited data on medical history and long-term changes, which may have an impact on a comprehensive understanding of the health status of older people in the community.

## 5. Conclusions

The results of this study show a correlation between the probable sarcopenia observed, low adherence to the MeDi, and cognitive impairment in the elderly population. These results build on the existing evidence of the association between cognition and sarcopenia in the elderly. These results should be considered when planning care for community-dwelling elderly and specialized institutions providing care for this group of patients could try to improve both cognitive and nutritional care in a multidisciplinary way.

## Figures and Tables

**Table 1:** Difference between two groups of participants according MMSE score.

	No (%) Population			*p* *
	MMSE ≥ 24 (*n* = 79)	MMSE < 24 (*n* = 35)	Total (*n* = 114)	
**Sex, *n* (%)**				0.89 *
Female	60 (75.9)	27 (77.1)	87 (76.3)	
Male	19 (24.1)	8 (22.9)	27 (23.7)	
**Age, years, median (IQR)**	79 (72–85)	84 (79–88)	81 (74–86)	0.01 ***
**Institution, *n* (%)**				
Stationary	27 (38.6)	18 (52.9)	45 (43.3)	0.17
Residential part	43 (61.4)	16 (47.1)	59 (56.7)	
**Age of entering the institution, median (IQR)**	72 (66–81)	80 (73–85.3)	76 (68.5–84)	<0.001 ***
**Duration, median (IQR)**	4 (0–11)	3 (0–5.3)	4 (0–8)	
**Marriage status, *n* (%)**				
Not married	17 (21.5)	4 (11.4)	21 (18.4)	0.02 *
Married	17 (21.5)	1 (2.9)	18 (15.8)	
Divorced	7 (8.9)	4 (11.4)	11 (9.6)	
Widowers	38 (48.1)	26 (74.3)	64 (56.1)	
**Education level, *n* (%)**				
Less than high school	19 (24.4)	21 (60)	40 (35.4)	0.001 *
High school graduate	42 (53.8)	10 (28.6)	52 (46)	
College graduate	17 (21.8)	4 (11.4)	21 (18.6)	
**Previous job activity, *n* (%)**				
Sedentary life	25 (32.1)	4 (11.4)	29 (25.7)	0.03 *
Less physical activity	33 (42.3)	15 (42.9)	48 (42.5)	
Heavier physical activity	20 (25.6)	16 (45.7)	36 (31.9)	
**Smoker (previous, current), *n* (%)**	28 (35.4)	6 (17.1)	34 (29.8)	0.05 *
Smoking (*n* cigarettes/year), median (IQR)	111,690 (66,619.8–191,625)	324,120 (158,775–438,000)	131,400 (83,044.8–212,430)	0.01 ***
**Blood pressure variables**				
Arterial hypertension, *n* (%)	55 (69.6)	23 (65.7)	78 (68.4)	0.67 *
SBP, mmHg, median (IQR)	139 (122–154)	121 (110–133)	130.5 (116–151.3)	<0.001 ***
DBP, mmHg, median (IQR)	80 (74–89)	72 (66–78)	77 (71–86)	<0.001 ***
**Medications** (pc), median (IQR)	4 (3–8)	6 (5–8)	5 (3–8)	0.02 ***
**Laboratory parameter, median (IQR)**				
**Total cholesterol** (mmol/L)	4.8 (3.8–5.5)	5.4 (5–5.7)	5 (4.4–5.5)	0.05 ***
**Triglycerides** (mmol/L)	1.2 (1–1.5)	1.7 (1.3–3.2)	1.2 (1–1.7)	0.04 ***

*p*-values were obtained with * χ^2^ test; *** Mann–Whitney U test for non-parametric numerical data. Abbreviations: MMSE = Mini Mental State Examination; IQR = interquartile range; *n* = number; SBP = systolic blood pressure; DBP = diastolic blood pressure.

**Table 2:** The difference in anthropometric and body composition parameters between two groups of patients according to MMSE Score.

	Median (IQR)			*p* *
	MMSE ≥ 24 (*n* = 79)	MMSE < 24 (*n* = 35)	Total (*n* = 114)	
**Anthropometric Parameters**				
Weight (kg), median (IQR)	71.45 (62.28–86.63)	69.2 (61.7–85.1)	71.2 (62.28–85.35)	0.68
Height (cm), median (IQR)	163 (158–169)	162 (158–165)	163 (158–168)	0.49
BMI (kg/m^2^), median (IQR)	26.75 (23.33–30.88)	27.6 (23.4–32)	26.8 (23.4–31.2)	0.76
Middle upper arm circumference (cm), median (IQR)	30 (26.5–34)	29.5 (26–32)	30 (26.3–33)	0.38
Waist circumference (cm), median (IQR)	97 (87.8–108.5)	100 (88.8–108.5)	99 (88–108)	0.43
WHtR, median (IQR)	0.6 (0.5–0.7)	0.6 (0.6–0.7)	0.6 (0.5–0.7)	0.17
**Body Composition**				
Fat mass (kg), median (IQR)	25.1 (15–31.5)	25.6 (14.5–30.6)	25.4 (14.9–31.2)	0.61
Fat-free mass (kg), median (IQR)	46.2 (41.8–53.9)	47.4 (43.5–54.1)	46.7 (42.5–53.9)	0.57
Bone mass (kg), median (IQR)	2.4 (2.1–2.7)	2.4 (2.2–2.7)	2.4 (2.2–2.7)	0.60
Predicted muscle mass (kg), median (IQR)	43.9 (39.7–51.2)	45 (41.3–51.4)	44.3 (40.3–51.2)	0.57
Total body water (kg), median (IQR)	32.4 (29.1–37.7)	33.1 (30.5–36.8)	32.8 (29.6–37.6)	0.68
Phase angle, median (IQR)	4.5 (3.9–4.8)	4.4 (3.9–5.1)	4.5 (3.9–4.8)	>0.99
ASM/ht^2^	6.5 (0–7.6)	7 (6.2–7.7)	6.8 (0–7.6)	0.24
Metabolic age (years), median (IQR)	69 (63–73.3)	71 (67–75.3)	70 (64–75)	0.17
TRFATM (kg), median (IQR)	11.8 (8.1–14.9)	12.2 (7.8–14.5)	12 (8–14.6)	0.76

* *p*-values were obtained with the Mann–Whitney U test for non-parametric numerical data. Abbreviations: MMSE = Mini Mental State Examination; BMI = Body mass index; WHtR = Waist-to-Height Ratio; IQR = interquartile range; ASM/ht^2^ = appendicular skeletal muscle mass/height^2^; TRFATM = Trunk Fat Mass.

**Table 3:** Differences in sarcopenia-predicting parameters between two groups of patients based on MMSE score.

	*n* (%) Population			*p* *
	MMSE ≥ 24 (*n* = 79)	MMSE < 24 (*n* = 35)	Total (*n* = 114)	
**SPPB Total Score**				<0.001 *
Low physical ability	24 (36.4)	29 (87.9)	53 (53.5)	
Moderate physical ability	19 (28.8)	2 (6.1)	21 (21.2)	
High physical ability	23 (34.8)	2 (6.1)	25 (25.3)	
**Muscle strength**				
Muscle strength (kg)/ fist grip test—dynamometer (medium value)	20.3 (15.3–26)	14 (9.3–20)	18 (11.6–24.8)	<0.001 **
Predicted muscle mass (kg)	43.9 (39.7–51.2)	45 (41.3–51.4)	44.3 (40.3–51.2)	0.57 **
**MNA (total score),** median (IQR)	25 (20.75–28)	23.5 (19–27)	24.75 (19.75–27.5)	0.01 **
**MNA (distribution, pc)**, *n* (%)	77	35		0.003 *
Normal nutritional status (24–30)	52 (67.5)	17 (48.6)	69 (61.6)	
At risk of malnutrition (17–23.5)	9 (11.7)	14 (40)	23 (20.5)	
Malnourished (<17)	16 (20.8)	4 (11.4)	20 (17.9)	
**SARC-F predictive of sarcopenia**				0.006 *
No	52 (67.5)	16 (48.5)	68 (61.8)	
Yes (≥4)	25 (32.5)	17 (51.5)	42 (38.2)	
**SARC-F (pc), *n* (%)**				0.02 *
No difficulty lifting/carrying 4.5 kg	45 (58.4)	13 (39.4)	58 (52.7)	
Sometimes difficulty lifting/carrying 4.5 kg	9 (11.7)	11 (33.3)	20 (18.2)	
Often difficulty lifting/carrying 4.5 kg	23 (29.9)	9 (27.3)	32 (29.1)	

*p*-values were obtained with * χ^2^ test; ** Mann–Whitney U test; Abbreviations: MMSE = Mini Mental State Examination; SPPB = Short Physical Performance Battery test; MNA = Mini Nutritional Assessment; SARC-F = the strength, assistance with walking, rising from a chair, climbing stairs, and falls questionnaire.

**Table 4:** (**a**) Statistically significant positive correlations between MMSE score and other variables. (**b**) Statistically significant negative correlations between MMSE score and other variables.

**(a)**	
	**Spearman’s Correlation Coefficient Rho (*p* Value)**
	**Total Score MMSE**
**Muscle strength** (kg)	0.438 (<0.001)
**Blood pressure—systolic** (mmHg)	0.364 (<0.001)
**Blood pressure—diastolic** (mmHg)	0.254 (0.01)
**Upper arm circumference** (cm)	0.342 (0.05)
**MNA total score**	0.206 (0.03)
**TMT—A** (min)	0.326 (<0.001)
**TMT—A** (s)	0.326 (<0.001)
**TMT—B** (min)	0.528 (<0.001)
**TMT—B** (s)	0.517 (<0.001)
**SDMT** (*n*)	0.569 (<0.001)
**MDSS**	
Potato intake adherence	0.205 (0.03)
Milk and dairy products adherence	0.185 (0.05)
Poultry adherence	0.310 (<0.001)
Red meat adherence	0.258 (0.01)
**(b)**	
	**Spearman’s Correlation Coefficient Rho (*p* Value)**
	**Total MMSE Score**
**Age**	−0.286 (<0.001)
**Age of entering the institution**	−0.306 (<0.001)
**Medications** (pc)	−0.218 (0.03)
**MDSS total score**	−0.177 (0.06)
Wine (quantity: 1 glass for women, 1–2 glasses for man)	−0.198 (0.03)

Abbreviations: MMSE = Mini Mental State Examination; MNA = Mini Nutritional Assessment; SDMT = Symbol Digit Modalities Test; MDSS = Mediterranean Diet Serving Score; min = minute; s = seconds; *n* = number.

**Table 5:** Predictors significantly impacting cognitive deficit reduction.

Bivariate Logistic Regression	ß	_Wald	*p* Value	OR	95% CI
**Age**	0.07	8.54	0.004	1.07	1.02–1.12
**Age of entering the institution**	0.05	7.39	0.007	1.05	1.01–1.09
**Marriage status** (Unmarried)					
Married	−1.46	1.65	0.20	0.23	0.02–2.16
Divorced	1.23	3.03	0.08	3.42	0.86–13.67
Widowed	1.18	4.69	0.03	3.25	1.12–9.46
**Education level** (Low education level)					
≤12 years	−1.49	12.86	0.003	0.22	0.09–0.51
>12 years	−1.72	9.21	0.002	0.18	0.06–0.54
**Activity** (Sedentary)					
Heavier physical activity	1.83	10.57	0.001	6.26	2.07–18.9
**Smoker** (yes)	−1.09	5.62	0.02	0.33	0.13–0.83
**Loss of appetite** (yes)	0.80	3.97	0.04	2.24	1.01–4.94
**Medications** (pc)	0.17	7.87	0.005	1.19	1.05–1.34
**Muscle strength** (kg)/ fist grip test—dynamometer (medium value)	−0.08	10.03	0.002	0.92	0.87–0.97
**SBP** (mmHg)	−0.03	9.74	0.002	0.96	0.94–0.98
**DBP** (mmHg)	−0.04	5.29	0.02	0.96	0.93–0.99
**SPPB** (Low physical activity)					
Moderate physical activity	−2.48	9.91	0.002	0.08	0.02–0.39
High physical activity	−2.62	11.17	0.001	0.07	0.02–0.34
**MNA** (Malnourished), score					
Malnutrition Risk	1.365	8.02	0.005	3.87	1.52–9.89
Normal nutritional status	0.28	0.42	0.52	1.32	0.57–3.06
**Predictive Sarcopenia** (≥4)	1.13	9.01	0.003	3.1	1.48–6.49
**Triglycerides** (mmol/L)	1.12	4.46	0.03	3.05	1.08–8.6
**SDMT, score**	−0.13	20.76	<0.001	0.88	0.83–0.93
**MDSS total score**	0.09	1.96	0.16	1.1	0.96–1.26
Eggs	−0.75	8.02	0.005	0.47	0.28–0.79
Poultry (chicken, turkey)	−2.13	8.19	0.004	0.12	0.03–0.51
ß—regression coefficients					

Abbreviations: SBP = Systolic Blood Pressure; DBP= Diastolic Blood Pressure; SPPB = Short Physical Performance Battery test; MNA = Mini Nutritional Assessment; SDMT = Symbol Digit Modalities Test; MDSS = Mediterranean Diet Serving Score.

**Table 6:** Predictors with significant impact on MMSE score (adjusted for age and gender).

	ß	Wald	*p* Value	OR	95% CI
**Multivariate logistic regression**					
**Gender**	−0.828	0.155	0.69	0.44	0.007–26.97
**Age**	0.829	5.47	0.02	2.29	1.14–4.59
**Predictive Sarcopenia (≥4) SARC-F**	5.87	4.08	0.04	353.6	1.19–105,308.2
**Constant**	−83.8	5.74	0.02		

ß—regression coefficients. Abbreviations: SARC-F = the strength, assistance with walking, rising from a chair, climbing stairs, and falls questionnaire.

## Data Availability

The raw data can be provided by the corresponding author via e-mail: josiparadic1973@gmail.com.

## Supplementary Materials

The following supporting information can be downloaded at https://www.mdpi.com/article/10.3390/nu16070991/s1, Table S1. Correlations of Mediterranean diet adherence and Mini-Mental State Exam (MMSE). Table S2. Predictors of Cognitive deficit—parts of Mediterranean Diet Serving Score. Table S3. Cognitive test performance differences between two groups of patients categorized by MMSE score. Table S4. Statistically significant positive correlations between MMSE score and other variables.
