# Supplementary material for: Unlocking Cognitive Potential: Association of Sarcopenia and Mediterranean Diet on Cognitive Function in Community-Dwelling Elderly of the Dalmatian Region
Source: Nutrients. 2024 Mar 28;16(7):991. doi: 10.3390/nu16070991 (PMC11013823; doi:10.3390/nu16070991)
Supplement: Supplementary file 1 [file nutrients-16-00991-s001.zip › nutrients-2929237-supplementary.pdf]

**Table S1.** Correlations of Mediterranean diet adherence and Mini-Mental State Exam (MMSE).

| | Sperman's correlation coefficient Rho (*p* value) |
|---|---|
| | Total score MMSE |
| Grains (including bread, rice, and pasta) | 0.143 (0.13) |
| Olive oil (including preparation of food) | −0.053 (0.57) |
| Nuts | 0.052 (0.58) |
| Fresh fruits (excluding fruit juices) | −0.073 (0.44) |
| Vegetables | 0.046 (0.63) |
| Legumes | 0.038 (0.69) |
| Eggs | 0.160 (0.09) |
| Fish (white and blue) | −0.069 (0.47) |
| Sweets (sugar, cakes, cookies, and fruit sodas) | −0.068 (0.48) |

**Table S2.** Predictors of cognitive deficit—parts of Mediterranean Diet Serving Score.

| | **ß** | ***Wald*** | ***p* Value** | **OR** | **95% CI** |
|---|---|---|---|---|---|
| Potato | −0.55 | 3.77 | 0.05 | 0.58 | 0.33–1.01 |
| Red meat (pork, beef, and veal) | −0.52 | 3.85 | 0.05 | 0.59 | 0.35–0.99 |
| Wine (in quantity: 1 glass for women, 1–2 glasses for man) | 18.5 | 0 | 0.99 | - | - |
| **MDSS total score** | 0.09 | 1.96 | 0.16 | 1.1 | 0.96–1.26 |

ß—regression coefficients.

**Table S3.** Cognitive test performance differences between two groups of patients categorized by MMSE score.

| | Median (IQR) | | | *p* * |
|---|---|---|---|---|
| | No cognitive decline (n = 79) | Moderate/Severe decline (n = 35) | Total (n = 114) | |
| **TMT – A** (s) | 49.1 (0–80.9) | 59.5 (33.72–90) | 49.2 (0–81.99) | <0.001 |
| **TMT - B** (s) | 46.5 (0–96.5) | 67.8 (38.4–120) | 54 (0–100.2) | <0.001 |
| **SDMT** (n) | 6 (0–20) | 12 (0–24.5) | 8 (0–20) | <0.001 |

* *p*-values were obtained with Mann–Whitney U test.

**Table S4.** Statistically significant positive correlations between MMSE score and other variables.

| | Spearman's correlation coefficient Rho (*p* value) |
|---|---|
| | Total score MMSE |
| **Muscle strength** (kg) | 0.438 (<0.001) |
| **Blood pressure—systolic** (mmHg) | 0.364 (<0.001) |
| **Blood pressure—diastolic** (mmHg) | 0.254 (0.01) |
| **Upper arm circumference** (cm) | 0.342 (0.05) |
| **MNA total score** | 0.206 (0.03) |
| **TMT – A** (min) | 0.326 (<0.001) |
| **TMT – A** (s) | 0.326 (<0.001) |
| **TMT – B** (min) | 0.528 (<0.001) |
| **TMT – B** (s) | 0.517 (<0.001) |
| **SDMT,** (n) | 0.569 (<0.001) |
| Potato | 0.205 (0.03) |
| Milk and dairy products | 0.185 (0.05) |
| White meat (chicken and turkey) | 0.310 (<0.001) |
| Red meat (pork, beef, and veal) | 0.258 (0.01) |

| | |
|---|---|
| **SF-36 quality of life** | |
| Physical functioning | 0.279 (<0.001) |
| Role limitation due to physical problems | 0.238 (0.01) |
| Mental health | 0.228 (0.02) |
| Energy vitality | 0.309 (<0.001) |
| Pain | 0.226 (0.02) |
| General health perception | 0.262 (0.01) |
| Change in health | 0.198 (0.04) |
